# Association Between Essential Trace Elements and Thyroid Antibodies in the Blood of Women with Newly Diagnosed Hashimoto’s Thyroiditis

**DOI:** 10.5812/ijem-145599

**Published:** 2024-08-12

**Authors:** Rahim Rostami, Asghar Beiranvand, Sarmad Nourooz-Zadeh, Massoumeh Rostami, Afshin Mohammadi, Jaffar Nourooz-Zadeh

**Affiliations:** 1 Department of Clinical Biochemistry, Iran University of Medical Sciences, Tehran, Iran; 2 Department of Epidemiology, Urmia University of Medical Sciences, Urmia, Iran; 3 Faculty of Medicine, Urmia University of Medical Sciences, Urmia, Iran; 4 Faculty of Medicine, Semnan University of Medical Sciences, Semnan, Iran; 5 Department of Radiology, Urmia University of Medical Sciences, Urmia, Iran; 6 Urmia University of Medical Sciences, Urmia, Iran

**Keywords:** Hashimoto Thyroiditis, Thyroid Antibodies (TPO-Ab, Tg-Ab), Selenium, Iron, Zinc, Copper

## Abstract

**Background:**

The involvement of essential trace elements in the pathogenesis of Hashimoto’s thyroiditis (HT) has been suggested, although the available evidence is limited.

**Objectives:**

The aim of this study was to investigate the interplay between serum selenium (Se), iron (Fe), zinc (Zn), and copper (Cu) status with thyroid auto-antibodies and thyroid echogenicity in women with newly diagnosed HT.

**Methods:**

A cohort of newly diagnosed female HTs (n = 56) and matched controls (n = 64) were recruited. Serum Se, Fe, Zn, and Cu were measured by furnace graphite atomic absorption spectrometry (FGAAS). Thyroid hormone profiles and thyroid autoantibodies were assessed via ELISA.

**Results:**

In HTs, mean serum Se, Fe, and Zn were significantly lower, while serum Cu was higher in HTs compared to controls (64.11 ± 20.75 vs. 92.3 ± 29.36 μg/L, 53.67 ± 14.09 vs. 70.38 ± 18.44 μg/dL, 64.38 ± 18.88 vs. 90.89 ± 29.99 μg/dL, and 101.18 ± 33.40 vs. 82.2 ± 26.82 μg/dL; all P < 0.001). Pearson correlation analysis revealed a significant inverse correlation between serum Se, Zn, and Cu with thyroid peroxidase antibody (TPO-Ab) and thyroglobulin antibody (Tg-Ab) levels (P < 0.001). While no significant correlation was observed between thyroid antibodies and serum Fe levels, logistic regression revealed associations between thyroid antibodies and serum Fe. Upon dividing serum Se and Zn into quartiles, there was a significant alteration in the levels of TPO-Ab and Tg-Ab, with a reduction in the levels of antibodies observed from the first quartile to the fourth quartile.

**Conclusions:**

We conclude that Se, Fe, and Zn deficiency, coupled with increased Cu levels, are associated with elevated thyroid antibodies in the setting of Hashimoto Thyroiditis.

## 1. Background

Hashimoto’s thyroiditis (HT) is the most prevalent cause of hypothyroidism in iodine-sufficient regions and is considered a risk factor for miscarriage, prematurity, coronary heart disease, and thyroid cancer (1). With a global annual incidence of 0.3 - 1.5 cases per 1000 individuals, women are at a 5 - 10 fold increased risk of developing HT compared to men (2-4). Hashimoto’s thyroiditis is characterized by elevated thyroid-stimulating hormone (TSH), anti-thyroid peroxidase (TPO-Ab), and anti-thyroglobulin (Tg-Ab) antibodies, accompanied by lymphocytic and plasmacytic infiltration of the thyroid parenchyma and the destruction of thyroid follicles (5, 6). The development of HT is influenced by both genetic and environmental factors, resulting in immunologic intolerance and the subsequent autoimmune reaction. A body of evidence suggests that trace element status is a key player in the development of HT, although the available literature is rather sparse and incongruent.

Essential trace elements are key for thyroid hormone synthesis and pivotal to thyroid autoimmunity (1, 7). Selenium (Se) exerts its effects on the thyroid mostly through selenoproteins, a large family of enzymes with significant influence on the regulation of oxidative balance and thyroid hormone synthesis (1). Iron (Fe), zinc (Zn), and copper (Cu) act as co-factors for thyroperoxidase (TPO), carboxypeptidase, deiodinases (types I and II), and antioxidant activity. Selenium, Fe, and Zn modulate the immune system through regulating cytokine synthesis and lymphocyte maturation (3, 8). Despite their general role in thyroid health and hormone production, the extent of their influence on the development of HT remains to be fully elucidated.

Stojsavljevic et al. reported an inverse correlation between serum Cu and Zn levels with the risk of autoimmune thyroiditis (AIT) (9). Liu et al. (10) also observed that higher serum Cu was associated with the grade of thyroid-specific antibodies [grade I: Negative TPO-Ab and Tg-Ab; grade II: Weak positive antibody group (TPO-Ab and/or Tg-Ab positive, and both < 500 IU/mL); and grade III: Strong positive antibody group (TPO-Ab and/or Tg-Ab ≥ 500 IU/mL)]. Rostami et al. (5) reported a correlation between Se deficiency with thyroid antibodies and enhanced oxidative stress in HTs. Other investigations have also revealed a link between iron deficiency (ID) and iron deficiency anaemia (IDA) with AIT and a higher prevalence of Thyroid peroxidase antibody (TPO-Ab) in women with severe to mild ID. In contrast, another study found no significant difference in trace element levels between AIT patients and healthy controls (11).

## 2. Objectives

Studies concentrating on the link between trace elements and thyroid autoimmunity are few and far between, with low sample sizes or the assessment of only a single trace element being their major limitation. To address these matters, this study aims to investigate the influence of serum Se, Fe, Zn, and Cu status on thyroid health and function in HTs.

## 3. Methods

### 3.1. Studied Population

A total of 172 participants were recruited from female patients referred for the first time to the endocrinology outpatient clinic of Imam Khomeini Teaching Hospital (Urmia, Iran), with the purpose of assessing thyroid health and function. The exclusion criteria for both groups were as follows: (1) history of chronic or recent (< 6 months) acute conditions (e.g., acute infections, stroke, myocardial infarction, and other cardiovascular diseases, diabetes, renal or hepatic impairment, autoimmune disease, bleeding disorders, cancer, etc.); (2) history of permanent or transient thyroid diseases; (3) multivitamin and/or trace element supplementation (since six months before enrollment); (4) menopause or irregular menstruation; (5) positive smoking history; (6) dieting or fasting; (7) current pregnancy and/or lactation; and (8) lack of consent to participate in the study.

Following a comprehensive assessment of thyroid antibodies, echogenicity, and function tests (TSH and fT4), 56 subjects were identified as being positive for both thyroid antibodies, hypo-echogenic, and exhibiting elevated TSH levels coupled with low fT4. Abnormal test results were defined as TPO-Ab (≥ 60 IU/mL), Tg-Ab (≥ 180 IU/mL), and elevated TSH levels (> 5.3 mIU/mL and < 0.7 ng/dL) (n = 56). The control group comprised (n = 63) subjects with normal TSH and being negative for anti-thyroid antibodies (TSH: 0.3 - 5.2 mIU/L and fT4: 0.7 - 1.8 ng/dL, respectively).

This investigation was approved by the ethics committee of Urmia University of Medical Sciences, Urmia, Iran (IR.umsu.rec.1388.32; IR.UMSU.REC.1398.027). All participants were informed of the study process and aims, and written informed consent was subsequently obtained from every individual or their legal guardian.

### 3.2. Blood Collection

Fasting blood samples (5 mL) were collected through venipuncture, kept at standard room temperature for 10 minutes, and subsequently centrifuged at 3500 RPM for 15 minutes. Serum aliquots (250 µL) were transferred to Eppendorf tubes and stored at -70°C until analysis.

### 3.3. Urine Collection

After providing adequate education on proper sample gathering technique, fasting urine samples (10 mL) were collected. Aliquots (1 mL) were transferred to Eppendorf tubes and kept at -70°C until analysis.

### 3.4. Thyroid Volume and Thyroid Echogenicity Assessment

To measure thyroid volume (TVol), a 7.5 MHz linear transducer real-time ultrasound instrument (Toshiba Nemio30, Japan) was used. Ultrasonography assessments were performed by a single senior radiologist on supine subjects while maintaining hyperextension of the cervical vertebrae. The following equation was used to determine the volume of each lobe: Width (cm) × length (cm) × depth (cm) × 0.479 (12). Total thyroid volume (TTVol) was calculated by adding up the volume of the left and right lobes. Thyroid tissue echogenicity was evaluated by comparing thyroid parenchyma with the adjacent cervical musculature (13).

### 3.5. Thyroid Function and Antibodies

Thyroid-stimulating hormone, T4, T3, fT4, and fT3 were assessed by ELISA (Pishtaz Teb, Tehran). Reference ranges, as well as inter- and intra-assay values, were adopted from the manufacturers’ instructions and are presented in Table 1. An enzyme immunoassay was used to determine TPO-Ab and Tg-Ab titres (EIA; AESKU Inc, Hamburg, Germany).

**Table 1.** Reference Ranges, Intra- and Inter-assay Variations ^a^

Parameter	Reference Range	Intra-assay Value (%)	Inter-assay Value (%)
**TSH (mIU/L)**	0.32 - 5.2	3.9 - 6	7 - 8.4
**T3 (ng/mL)**	0.6 - 2.1	3.1 - 3.8	3.6 - 8.8
**T4 (μg/dL)**	4.7 - 12.5	3.6 - 5.8	4.4 - 7.6
**fT3 (pg/mL)**	1.9 - 4.3	4.0 - 4.8	5.5 - 7.9
**fT4 (ng/dL)**	0.7 - 1.9	3.5 - 6	3.9 - 7.1
**Tg (ng/mL)**	2 - 50	5 - 10	8 - 20
**TPO-Ab (IU/mL)**		3 - 5	3.5 - 6.5
Negative	≤ 40		
Intermediate	40 - 59		
Positive	≥ 60		
**Tg-Ab (IU/mL)**		3.9 - 5.8	3.9 - 7.1
Negative	≤ 120		
Intermediate	120 - 179		
Positive	≥ 180		
**Serum Cu (μg/dL)**	70 - 140	4.1 - 6.5	4.9 - 7.6
**Serum Zn (μg/dL)**	58.2 - 120	3.1 - 5.9	4.5 - 6.7
**Serum Se (μg/L)**	67-121	3.5 - 5.7	4.8 - 7.1
**Serum Fe (μg/dL)**	60 - 160	3.9 - 6.1	4.9 - 7.5

Abbreviations: TSH, thyroid-stimulating hormone; Tg, thyroglobulin; TPO-Ab, thyroid peroxidase antibody; Tg-Ab, thyroglobulin antibody; Se, selenium; Fe, iron; Zn, zinc; Cu, copper.

^a^ Reference ranges, intra- and inter assay values.

### 3.6. Thyroglobulin Assay

Assessment of thyroglobulin (Tg) was performed using an enzyme immunoassay (EIA; AESKU Inc, Hamburg, Germany).

### 3.7. Serum Selenium, Zinc, Copper, and Iron Assay

Graphite-furnace atomic absorption spectrometry (GF-AAS) was used to determine serum Se, Fe, Zn, and Cu values. The GF-AAS was equipped with a hollow cathode lamp and pyrolytically coated graphite tubes (GF-AAS; PG 990, England). Pure Argon was used for measurement. For each element, signal reading was performed as follows: Zn: 213.9 nm at a slit width of 1.0 nm; Cu: 324.4 nm at a slit width of 1.3 nm; Se: 196.0 nm at a slit width of 0.7 nm; and Fe: 248.3 nm at a slit width of 1.0 nm (14-17). Trace element serum calibration was performed using Seronorm (Seronorm™, Sero, Billingstad, Norway).

### 3.8. Urinary Iodine Excretion Assessment

Urinary iodine excretion (UIC) assessment was carried out based on the Sandell-Kolthoff reaction, as described elsewhere (18, 19). Briefly, thawed urine samples were vortexed to release particulates and subsequently centrifuged at 15,000 RPM for 5 minutes. The urine samples were then mixed with an ammonium persulfate solution (1 to 4 proportion), heated at 100°C for 1 hour, and allowed to stand at room temperature for 10 minutes. To this, a cerium (IV) ammonium sulphate (VI) solution was then added. Immediately after mixing, absorbance was measured at 405 nm using a double beam UV/Vis Perkin Elmer spectrophotometer (PerkinElmer, USA).

### 3.9. Urinary Creatinine Assessment

Urinary creatinine was measured according to the Jaffe method (Pishtaz Teb, Tehran, Iran) using a BT-1500 auto-analyser, on 1/50 diluted urine samples.

### 3.10. Statistical Analysis

SPSS software package for Windows v.24 (IBM; USA) was used for statistical analysis. Quantitative data are expressed as means ± SD or medians. Numeric variables were analyzed using Student’s *t*-test or the Mann-Whitney U-test (for nonparametric data), while categorical data were analyzed using the χ^2^ test. Nonparametric data were natural logarithm-transformed when possible. Pearson’s correlation coefficient and the Spearman rank correlation coefficient were used to evaluate associations between variables with normal and non-normal distribution patterns, respectively. Tukey’s HSD test was used to assess the significance of differences observed between the two groups regarding thyroid function tests, volume, age, autoantibodies, Se, Fe, Cu, and Zn levels. Logistic regression analysis (forward and backward stepwise) was used to examine the relationship between thyroid antibodies and serum trace elements, which are presented as odd ratios (ORs) and 95% confidence intervals (95% CIs).

## 4. Results

### 4.1. Patient and Control Characteristics

**Figure 1.**
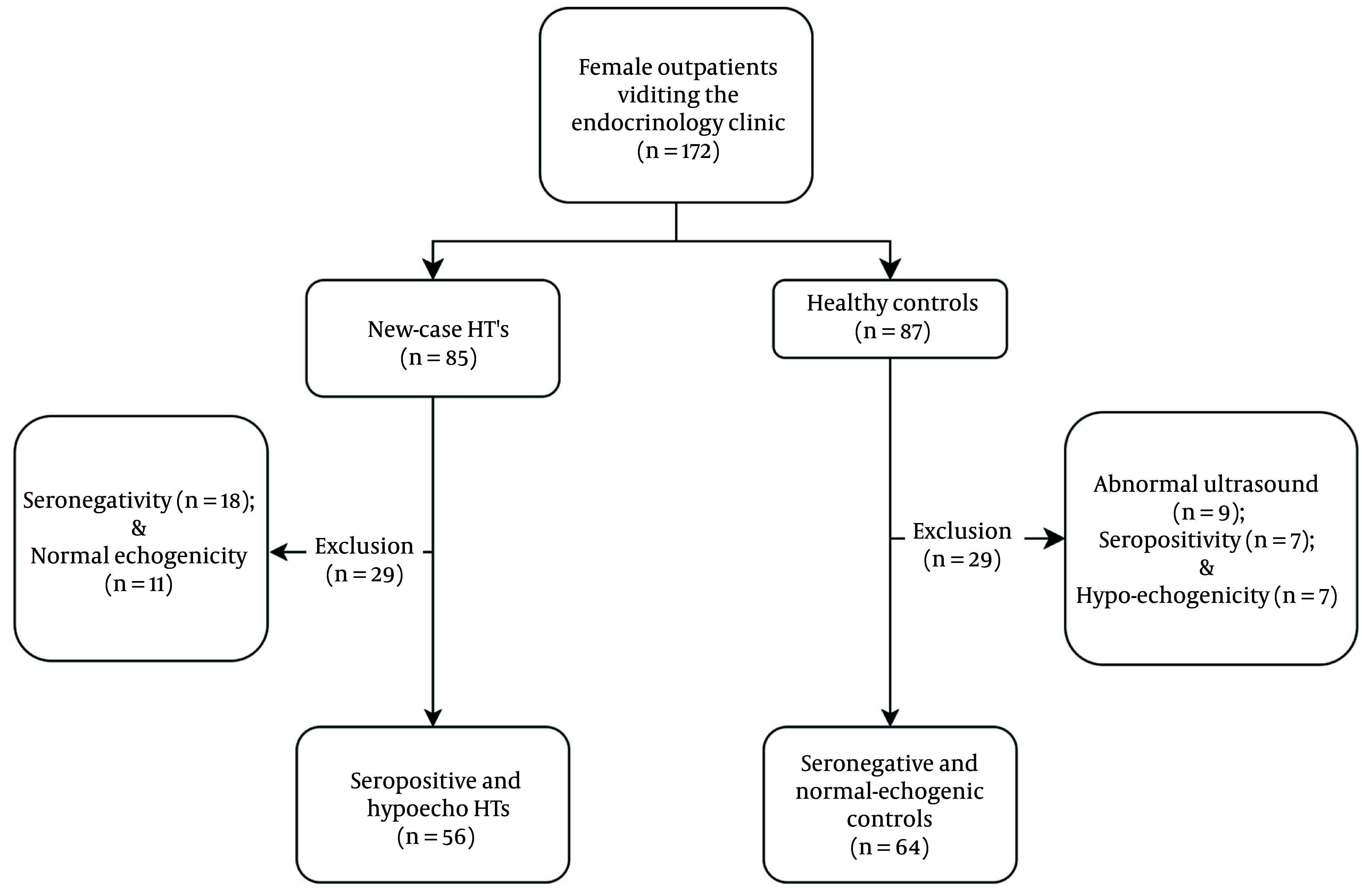
Patient and control enrolment process flowchart

The enrollment flowchart is presented in Figure 1. The mean age of the study pool was 33.28 ± 11.54 years. The mean age of the HTs and controls was 33.5 ± 10.2 and 33.7 ± 10.7 years, respectively. Table 2 summarizes the demographic and clinical values by groups (HT vs. control). No significant difference was observed between the groups regarding anthropometric measures, except for Body Mass Index (BMI), which was slightly higher in the HTs (28.93 ± 8.44 vs. 25.31 ± 5.31 kg/m²; P = 0.022).

**Table 2.** Clinical Biomarkers and Demographic Data of Hashimoto’s Thyroiditis and Controls

Parameters	Hashimoto Thyroiditis (n = 56)	Controls (n = 64)	P-Value
**Age (old years)**	33.50 ± 10.17	33.69 ± 10.68	0.836
**BMI (kg/m** ^**2**^ **)**	28.93 ± 8.44	25.31 ± 5.31	0.022
**Iodinated salt (ppm)**	20.12 ± 9.53	21.23 ± 8.83	0.335
**TSH (mIU/L)**	18.57 ± 12.37	1.41 ± 1.15	0.001
**T4 (μg/dL)**	6.53 ± 2.28	8.94 ± 1.61	0.001
**T3 (ng/ml)**	1.23 ± 0.41	1.94 ± 0.45	0.001
**fT4 (ng/dL)**	0.70 ± 0.37	1.15 ± 0.29	0.000
**fT3 (pg/mL)**	2.96 ± 0.76	3.59 ± 0.61	0.001
**Tg (ng/mL)**	22.04 ± 17.50	10.8 ± 11.81	0.001
**TVol (mL)**	17.41 ± 8.50	9.80 ± 4.14	0.001
**Anti-TPO (U/mL)**	474.5 ± 323.6	4.54 ± 5.60	0.001
**Anti-Tg (U/mL)**	787.3 ± 843.3	17.31 ± 22.61	0.001
UIC* (μg/L)	166.1 ± 130.9	132.8 ± 108.9	0.104
**UIC/Cr ratio (μg/mg)**	196.3 ± 172.2	75.8 ± 72.3	0.001
**Zn (μg/dL)**	53.67 ± 14.09	70.38 ± 18.44	0.001
**Cu (μg/dL)**	101.18 ± 33.40	82.2 ± 26.82	0.001
**Se (μg/L)**	64.11 ± 20.75	92.3 ± 29.36	0.001
**Fe (μg/dL)**	64.38 ± 18.88	90.89 ± 29.99	0.001
**Ferritin (ng/mL)**	22.71 ± 14.75	39.63 ± 30.99	0.003

Abbreviations: BMI, Body Mass Index; TSH, thyroid-stimulating hormone; Tg, thyroglobulin; TVol, thyroid volume; UIC, urinary iodine excretion; Se, selenium; Fe, iron; Zn, zinc; Cu, copper.

In the HTs, serum Se, Fe, and Zn levels were found to be lower (P < 0.001) compared to the control group. Conversely, Cu levels were higher in HTs than in the control group (P < 0.05). Based on a cut-off value of < 58.2 μg/dL, it was discovered that 58% of the HTs and 31% of the controls were Zn deficient. The respective values for Se deficiency were 57.1% of the HTs and 30.2% of the controls. Iron deficiency was found in 35.7% and 15.6% of the HTs and controls, respectively. Furthermore, Cu deficiency was observed in 27% and 44% of the HTs and controls. It was also noted that 60% and 13% of HTs exhibited sufficient and toxic levels of serum Cu, respectively. In the control group, these values were 51% and 5%, respectively.

**Figure 2.**
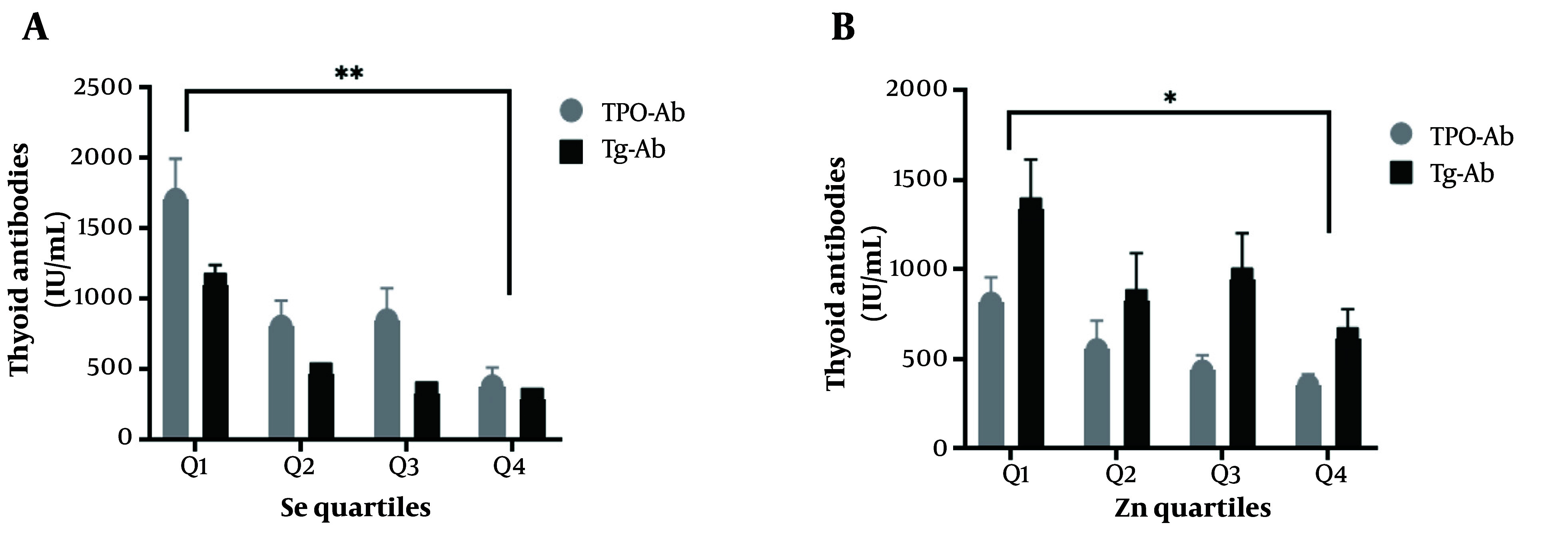
Variation in the levels of thyroid peroxidase antibody (TPO-Ab) and thyroglobulin antibody (Tg-Ab) in selenium (A); and zinc (B) quartiles

Based on the data presented in Figure 2, Se and Zn were divided into quartiles. The study revealed that HTs with the highest levels of Se and Zn (fourth quartile) had notably lower TPO-Ab and Tg-Ab levels than those in the first quartile. However, no significant difference in the levels of thyroid antibodies was observed with regards to Fe and Cu quartiles.

T4, T3, fT3, and fT4 levels were found to be lower in HTs than in the controls (all P < 0.05), while Tyg, TSH, TPO-Ab, Tg-Ab, TVol, and UIC/Cr were higher in the HTs (all P < 0.05). All laboratory biomarkers and demographic data have been presented in Table 2. 

### 4.2. Association of Serum Trace Elements and Thyroid Antibodies

**Figure 3.**
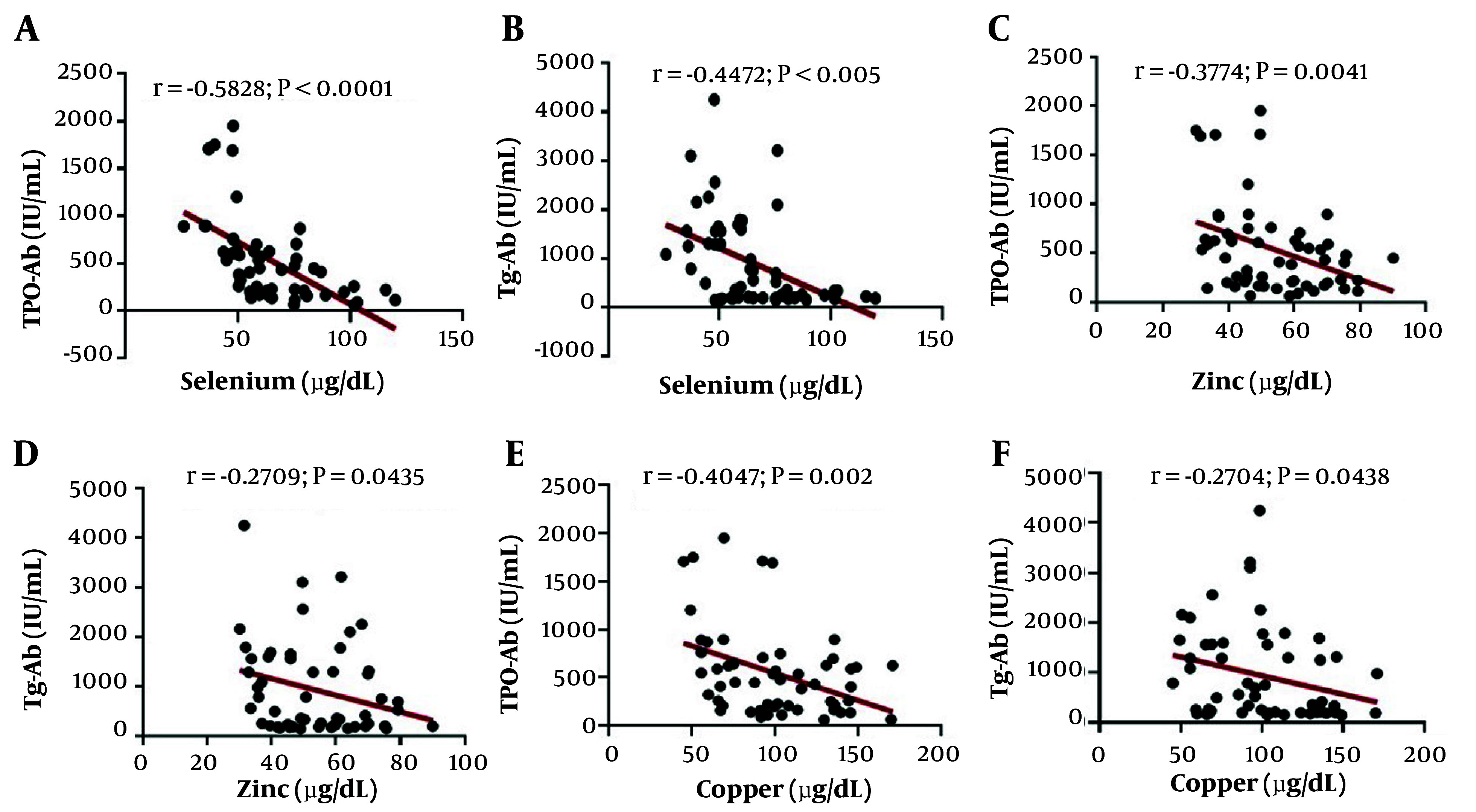
Correlation between essential trace elements and thyroid antibodies in Hashimoto’s thyroiditis (HT). A, B, C, Spearman rank correlation analysis was used; D, E, F, Pearson’s correlation coefficient was used.

In HTs, significant associations were observed between Se, Zn, and Cu levels with TPO-Ab and Tg-Ab (all P < 0.05). Binary logistic regression analysis was carried out for each element, with due consideration to adjustments for covariates. It was found that trace elements are positively correlated with HT based on the model adjusted for age and BMI (Se: OR = 1.038, 95% CI: 1.014 - 1.062, P = 0.001; Fe: OR = 1.049, 95% CI: 1.018 - 1.082, P = 0.002; Zn: OR = 1.051, 95% CI: 1.015 - 1.087, P = 0.005; Cu: OR = 0.971, 95% CI: 0.950 - 0.992, P = 0.007; and UIC/Cr ratio: OR = 0.990, 95% CI: 0.980 - 0.999, P = 0.00021). The association between serum trace elements and thyroid antibodies is shown in Figure 3. 

## 5. Discussion

This study explored the association between thyroid antibodies and serum essential trace elements, namely Se, Fe, Zn, and Cu. We found that TSH, TVol, Tg, TPO-Ab, Tg-Ab levels, and UIC/Cr ratio were significantly higher in HTs than in controls, whereas the levels of T4, T3, fT4, fT3, and ferritin were significantly lower in the former.

Pearson correlation analysis revealed that in HTs, Se, Zn, and Cu were inversely associated with TPO-Ab and Tg-Ab levels (Figure 3). However, regarding Fe and iodine, no correlation was observed. Binary regression analysis revealed that serum Se, Fe, Zn, Cu levels, and UIC/Cr ratio exhibited a non-linear correlation with HT and TPO-Ab and Tg-Ab. At the same time, no association was detected between trace elements and thyroid antibodies in the controls.

Selenium is an essential trace element found in various grains, meats, and eggs, among other things. Despite this, Se deficiency is common worldwide due to inadequate resources and has been linked to an increased risk of autoimmune disorders by a number of population-based studies. The thyroid gland contains high concentrations of Se, mostly in the form of selenoproteins, such as glutathione peroxidase (GPx3) and deiodinases. These proteins protect the thyroid from the oxidative stress inflicted by thyroid hormone synthesis and other reactions occurring in the thyroid gland (5). Moreover, the incorporation of Se as selenocysteine into the structures of selenoproteins plays a crucial role in the endoplasmic reticulum quality-control machinery of newly synthesized thyroid proteins, providing another direct link between Se intake, Se status, and the interaction of thyrocytes with the immune system, with potential relevance for autoimmune thyroid diseases (AITD) (20).

In the present study, Se levels were significantly decreased in HTs compared to controls. We divided Se levels into quartiles and observed that TPO-Ab and Tg-Ab in the fourth quartile were significantly lower than in other quartiles. In other words, subjects with higher levels of Se had lower levels of thyroid antibodies (Figure 2). Furthermore, serum Se had an inverse association with TPO-Ab and Tg-Ab. In a population-based study, Wu et al. found that the incidence of HT in individuals with low-Se levels was 3.65-fold higher compared to people with high serum Se, suggesting the deficiency of this trace element as a risk factor for the development of HT (20). These findings were supported by a study by Wu et al., which indicated higher serum Se levels were associated with a low incidence of AIT, hypothyroidism, subclinical hypothyroidism, and thyroid enlargement (OR = 0.47, 0.75, 0.63, 0.68, and 0.75, respectively) (21). Moreover, Se supplementation for 3 - 12 months reduced TPO-Ab and Tg-Ab titres in patients with HT, while levothyroxine (LT4) therapy was found to only impact TPO-Ab titres (22, 23). A proposed molecular mechanism for the biological function of Se in modulating the immune system involves the role of selenoproteins, specifically thioredoxin reductases and selenoproteins K, which are crucial in regulating T cell maturation, modulating inflammation, and cytokine production (24).

The results of this study showed that Fe levels were lower in HTs compared to the control group. Although we did not observe a linear correlation between thyroid antibodies and Fe levels, logistic regression analysis revealed a non-linear relationship between thyroid antibodies and serum Fe. Luo et al. (1) found that HTs had significantly lower Fe levels compared to healthy individuals. Despite the lack of a direct correlation between Fe levels and thyroid antibodies (TPO-Ab and Tg-Ab) based on the Pearson correlation analysis, more complex binary logistic regression methods reveal a nonlinear relationship, suggesting Fe's potential role as an independent protective factor against thyroid autoantibodies. This is supported by studies reporting significant differences in serum Fe concentrations between individuals positive for thyroid antibodies compared to those without, suggesting a nonlinear association which underscores the importance of Fe in thyroid health (1, 25-28)

Iron plays a crucial role in maintaining thyroid function and hormone synthesis, with deficiencies leading to reduced activity in key enzymes and impairments in hormone conversion processes (27, 29). Notably, ID has been linked to an increased risk of AIT, particularly among pregnant women and individuals with thyroid dysfunction (27, 28, 30). The relationship between ID and thyroid autoimmunity may be attributed to changes in the antigenicity of TPO, as well as the reduced activity of TPO in the context of Fe deficiency (25-28). Additionally, adequate Fe intake is essential for proper immune function, potentially offering protection through the enhancement of Th1 activity, despite the ongoing debate over the implications of Fe overload on oxidative stress (31, 32).

It was found that Zn was significantly lower in HTs compared to controls. Furthermore, a significant correlation was observed between Zn status with TPO-Ab and Tg-Ab (P<0.05). When Zn levels were divided into quartiles, TPO-Ab and Tg-Ab in quartile four were significantly lower than other quartiles (Figure 2). In a cross-sectional study, Ertek et al. (33) reported that serum Zn significantly correlated with TPO-Ab levels in AITD subjects. However, Borawska et al. revealed that anti-TPO titers were inversely correlated with Zn levels in HT women (34).

There is accumulating evidence indicating that Zn plays a key role in the regulation, synthesis, and release of various cytokines, as well as thymulin and lymphocyte activity (3). Moreover, Zn deficiency and hypothyroidism can negatively affect T cell count and increase apoptosis (35), possibly through causing an imbalance between Th1 and Th2 ratios, increasing Th17 lymphocyte count, and promoting T- and B cell-related pro-inflammatory activity. Together, these potentiate the development of AIT. Therefore, Zn supplementation (60 µM, three times per day) may suppress the risk of allogeneic reaction without affecting antigenic response (35-37).

Unlike serum Se, Fe, and Zn levels, Cu levels were significantly higher in HTs than controls, while also exhibiting a significant association with TPO-Ab and Tg-Ab in the former group. A body of evidence suggests that Cu contributes to thyroid hormone production through Fe transportation and the activation of TPO, maintaining proper thyroid hormone synthesis (38, 39). However, a high concentration of Cu may promote cancerous alteration by damaging DNA with toxic free hydroxyl radicals.

Thyroid hormones tightly regulate Cu levels, and T3 unregulates ceruloplasmin in response to Cu intake (38, 39). Information regarding the relationship between Cu and thyroid autoimmunity is limited and unclear. Liu et al. found that in hyperthyroid and AIT patients, Cu levels were elevated while also showing an association with TPO-Ab and Tg-Ab levels. Their results suggested that thyroid autoimmunity and hyperthyroidism may share a link with relatively high serum Cu levels (10). One possible explanation is that impaired serum Cu homeostasis may contribute to increased oxidative stress in HTs. Accumulation of Cu could also trigger the Fas/FasL (Fas ligand) signalling pathway and subsequently induce apoptosis in thyrocytes, which contributes to the pathogenesis of AIT (10).

There are some limitations in this study. Due to the nature of the study, we could not assess the causality between Se, Fe, Zn, and Cu with thyroid antibodies. Future studies should aim for longitudinal observation and mechanism interpretation, coupled with extensive assessment of other trace elements with previously suggested roles in maintaining thyroid health and function, such as calcium and magnesium. Controlled supplementation may also provide better insight into the mechanisms by which these elements influence the thyroid. Since the current study population resides in northwest Iran, a region with marginally sufficient iodine intake (with its possible independent influence on thyroid function), the findings of the current study may not be generalized to other regions due to both ethnic and geographic factors.

### 5.1. Conclusions

In conclusion, trace elements are essential in various physiological and pathological processes and are closely connected to thyroid health and function. Low Se, Zn, and Fe blood levels may be associated with thyroiditis-associated antibodies. After adjustments, Se, Zn, and Cu displayed an inverse linear correlation with thyroid antibodies, while Fe showed a non-linear correlation with thyroid antibodies. This study also suggests serum Cu levels could potentially be linked to HT. Our findings suggest monitoring serum trace element levels and supplementation with appropriate amounts of Se, Zn, and Fe may help maintain thyroid homeostasis and improve immune function.

## Data Availability

The dataset presented in the study is available on request from the corresponding author during submission or after publication.
